# RNAi turns 25:contributions and challenges in insect science

**DOI:** 10.3389/finsc.2023.1209478

**Published:** 2023-10-04

**Authors:** Subba Reddy Palli

**Affiliations:** Department of Entomology, Martin-Gatton College of Agriculture, Food and Environment, University of Kentucky, Lexington, KY, United States

**Keywords:** RNAi, dsRNA, endosomes, insects, pests, nanoparticles

## Abstract

Since its discovery in 1998, RNA interference (RNAi), a Nobel prize-winning technology, made significant contributions to advances in biology because of its ability to mediate the knockdown of specific target genes. RNAi applications in medicine and agriculture have been explored with mixed success. The past 25 years of research on RNAi resulted in advances in our understanding of the mechanisms of its action, target specificity, and differential efficiency among animals and plants. RNAi played a major role in advances in insect biology. Did RNAi technology fully meet insect pest and disease vector management expectations? This review will discuss recent advances in the mechanisms of RNAi and its contributions to insect science. The remaining challenges, including delivery to the target site, differential efficiency, potential resistance development and possible solutions for the widespread use of this technology in insect management.

## 1 Introduction

The target-specific interference of mRNA transcription, stability, and translation by complexes of Argonaute (Ago) family proteins and small RNAs such as small interfering RNA (siRNA) and microRNA (miRNA) resulting in decreased levels of gene products and their function is referred to as RNA interference (RNAi) (1, 2). Twenty-five years of research on methods development, mechanisms of action, and development of applications in animals, humans, and plants led to its widespread use in basic and applied research (3, 4). RNAi methods contributed significantly to advances in all areas of biology. RNAi-based therapeutics have been developed and are being used for the treatment of human diseases. Also, RNAi-based methods have been developed for pest and disease control and crop improvement in agriculture. Science Journal declared RNAi as the technology of the year in 2002. The Nobel Prize in Medicine for 2006 was awarded to Fire and Mello for discovering RNAi. Since its discovery, RNAi has played an important role in advances in insect science. Initial research on RNAi in insects was conducted in the model insect, *Drosophila melanogaster*. During the next few years, RNAi function was discovered in many insect species, including some economically important insects such as pests, disease vectors, and beneficial insects. RNAi works efficiently in some groups of insects, such as beetles but is inefficient in other insects, such as moths and butterflies. The differential efficiency of RNAi among pests and the potential development of resistance to RNAi are some of the major hurdles to the widespread use of RNAi in insect pest and disease vector management. There are many reviews published on RNAi; rather than adding one more to this list of reviews on this subject, I will focus my discussion on the critical evaluation of contributions of RNAi over the past 25 years along with challenges and possible solutions for widespread use of this technology for the management of pests and disease vectors.

## 2 RNAi discovery

Gene silencing research started with an unexpected phenotype detected in petunia plants. When a gene coding for an enzyme to enhance purple color was introduced into petunia plants, the transgenic plants showed a decrease rather than an increase in purple color (5). Further research revealed co-suppression of the endogenous gene coding for the enzyme involved in the production of purple color (6). Co-suppression of the endogenous gene was reported for virus resistance in plants and animals. La Crosse (LA) virus gene introduced into an infectious Sindbis virus expression vector caused interference to its replication (7). Also, the expression of virus genes interfered with the replication of the California serogroup virus in mosquito cells and mosquitoes (8). The silencing of genes was reported in the fungus, *Nuerospora crassa* (9). However, in these studies, the trigger of silencing and the mechanism of silencing were not identified. A landmark publication in RNAi by Fire and Mello (2) in 1998, for the first time, identified double-stranded RNA (dsRNA) as the molecule that triggered RNA silencing and coined the term RNA interference (RNAi). This publication also reported on the mechanism of action of dsRNA to achieve target gene silencing. Subsequent research during the next few years reported RNAi in nematodes, insects, plants, and trypanosomes (10–18). The functioning of RNAi was reported in mammalian cells in 2001 (19) and mice in 2002 (20). Research on RNAi during the past 25 years resulted in development of many applications in medicine and agriculture (21–28).

## 3 RNAi in insects

The first insect to demonstrate RNAi function was the fruit fly, *Drosophila melanogaster* (12, 29). Fruit fly genetic and genomic tools were employed to study the functions of genes using transgenic flies (30). Transgenic fly lines expressing RNAi triggers for almost all genes identified in the *Drosophila* genome are available from *Drosophila* stock centers. These UAS transgenic flies can be crossed with GAL4 lines expressing GAL4 in a tissue and stage-specific manner, allowing the knockdown of target genes in specific tissues and stages. The addition of RNAi to the *Drosophila* toolbox revolutionized functional genomics in this model insect and facilitated studies on the function of genes involved in almost every aspect of insect life (3). High throughput screening assays were developed using *Drosophila* cell lines and transgenic flies and used to identify genes and their function in developmental and physiological processes in flies (31–57). During the next few years functioning of RNAi was demonstrated in other insects such as *Anopheles gambiae* (30), *Tribolium castaneum* (18), *Anopheles stephensi* (22), *Blattella germanica* (58), *Manduca sexta* (59), *Apis mellifera* (60–62), *Aedes aegypti* (63), *Locusta migratoria* (64), *Leptinotarsa decemliata* (65) and *Nilaparvata lugens* (66).

## 4 RNAi mechanism

Double-stranded RNAs in the cytoplasm of the cells are recognized by dicer enzymes and digest them into small interference RNA (siRNA) of 18-21 base pair length (20, 67, 68). The RNA-Induced Silencing Complex (RISC) containing argonaute proteins (69, 70) facilitates the binding of siRNA to complementary DNA/mRNA blocking replication, transcription and translation (71). This results in reduced levels of target gene products hence the knockdown or knockout effect of the target gene function. In mammalian cells, dsRNAs induce interferon response (72). Most of the the critical players including, dicers and argonautes involved in RNAi response are conserved in insects. However proteins involved in dsRNA uptake and transport into cytoplasm vary among insect species contributing to differences in RNAi response among insect species (more discussion on this in section 6) However, the interferon response in insect cells is not robust. Therefore, long dsRNAs could be used as RNAi triggers in insects. The exogenously applied dsRNA is processed to multiple siRNAs by insect cells bypassing the need for optimization of siRNA for each gene normally required for efficient silencing of target genes in vertebrates. Long dsRNAs (200-400 bp) are delivered to insect cell lines *in vitro* or injected into insects *in vivo* to induce RNAi response. This makes it easier to implement RNAi technology in laboratories without access to bioinformatics tools. This may be one of the reasons for the extensive use of RNAi in entomology laboratories all over the world. RNAi technology contributed to advances in our knowledge of pest biology, insecticide targets and resistance development to insecticides. Most of this research on non-model insects during the past 25 years would not have been possible without the use of RNAi technology.

## 5 Applications of RNAi in basic science and pest control

RNAi made significant contributions to advances in our understanding of insect biology. A combination of genetics and genomic tools and the availability of genome sequences in model insects such as the fruit fly, silk moth and red flour beetle facilitated research in functional genomics to determine functions of genes involved in insect development, reproduction and behavior. RNAi also played an important role in advancing the biology of non-model insects that include pests, disease vectors and beneficial insects. Research on the identification and characterization of insecticide target sites and mode of action of insecticides and resistance development against insecticides by insects is aided by the use of RNAi methods. Functional genomics studies aided by RNAi in destructive pests such as the brown plant hopper, locust and disease vectors such as the yellow fever mosquito advanced our knowledge on development, reproduction and immune response in these insects.

RNAi works well and is used extensively in the red flour beetle, *Tribolium castaneum* to study the function of genes involved in growth, development, reproduction and insecticide resistance (73–100). The western corn rootworm, *Diabrotica virgifera virgifera* is another coleopteran insect where RNAi helped to identify targets for insecticide development (101, 102).

Expression of RNAi triggers in plants results in the knockdown of target genes in insects that feed on transgenic plants resulting in their mortality (101, 103). These results showed the potential of RNAi technology in pest management and attracted investment from both public and private sectors for the development of RNAi-based methods for controlling pests and disease vectors. The utility of RNAi technology to protect crops and trees from insect damage was tested in multiple systems, including cotton (104–106), rice (107), potato (108, 109), tobacco (110–113), poplar (114) and wheat (115). Surprisingly, the plant-mediated delivery of RNAi triggers was tested only in a few crop-pest systems. Difficulty in producing transgenic plants for economically important crops and hesitancy in public acceptance of food derived from genetically modified crops may have contributed to the slow progress of research in this area. Despite 20 years of efforts, only one plant-mediated RNAi product, corn seed for protection against corn rootworm has been commercialized.

In insects such as the Colorado potato beetle, tephritid fruit fly (116) and beet armyworm (117), feeding dsRNA produced in bacteria induces efficient knockdown of target genes and mortality. dsRNA produced in yeast (118) and algae (119–121) was also reported to trigger RNAi in mosquitoes. *In vitro* synthesized dsRNA also induces efficient RNAi in coleopteran insects such as the Colorado potato beetle and flea beetle, and these products are under development for registration to control these pests. RNAi is also being developed to protect beneficial insects from pathogens such as the Israeli Acute Paralysis Virus that infects honey bees (122). Remebee-1, a dsRNA product, was developed to control this disease of honey bees (60). Transgenic silkworms expressing dsRNA targeting *lef-1* gene of *Bombyx mori* nucleopolyhedrovirus (BmNPV) were developed to control this viral disease (123, 124). Another application of RNAi is for blocking the transmission of human and plant pathogens by insect vectors. dsRNA targeting the defensin gene in *Anopheles gambiae* adults reduced antimicrobial defense against Gram-positive bacteria (30). Also, knockdown of a *complement-like protein 1, TEP1*, gene in *An. gambiae* increased the number of developing parasites in the susceptible strain but abolished melanotic refractoriness in the refractory strain (125). RNAi played a critical role in understanding host-parasite interactions and immune responses in mosquitoes (126–140).

## 6 Roadblocks to widespread use of dsRNA pesticides

After 25 years of research on RNAi, why do we have only a few RNAi-based pesticides registered for pest management? The differential efficiency of RNAi among insects and its lower efficiency in major pests, potential resistance development and public hesitancy in accepting food from genetically modified crops are the major hurdles to the widespread use of RNAi in pest management. After discovering the functioning of RNAi in many insects, it became clear that RNAi works with variable efficiency among insect species tested. In beetles belonging to Tenebrionidae, Chrysomelidae and other families, the RNAi is efficient and systemic (141–147). Injection of dsRNA induces efficient RNAi in hemipteran insects, the milkweed bug (*Oncopeltus fasciatus*) (148), the brown marmorated stink bug (*Halyomorpha halys*) (149), the bed bug (*Cimex lectularius*) (150) and also in other insects such as *B. germanica* (58), *S. gregaria* and *L*. *migratoria* (151, 152) belonging to other orders. In contrast, RNAi is variable and inefficient in insects from most of the other orders, such as Lepidoptera (153). Interestingly, RNAi works well in leafhoppers from the order Hemiptera but is inefficient in aphids belonging to the same order. In most insects, injection works better than feeding for delivering dsRNA. In *Drosophila*, transgenic expression of hairpin dsRNA works more efficiently compared to feeding or injection of dsRNA. In locust and a few other insects only injection of dsRNA triggers RNAi; feeding dsRNA in these insects does not result in the knockdown of target genes (151). RNAi efficiency is also variable in different tissues and stages of an insect. RNAi efficiency also depends on the target gene and its expression levels (154). Constitutively and highly expressed target genes are knocked down much more efficiently than those expressed at lower levels and restricted developmental stages and tissues (154). These results suggest that degradation of RNA in the alimentary canal, delivery of dsRNA to cytoplasm for digestion by dicer enzymes and recruitment by RISC complex and expression levels of genes involved in RNAi might play important roles in determining RNAi efficiency.

**Figure 1:**
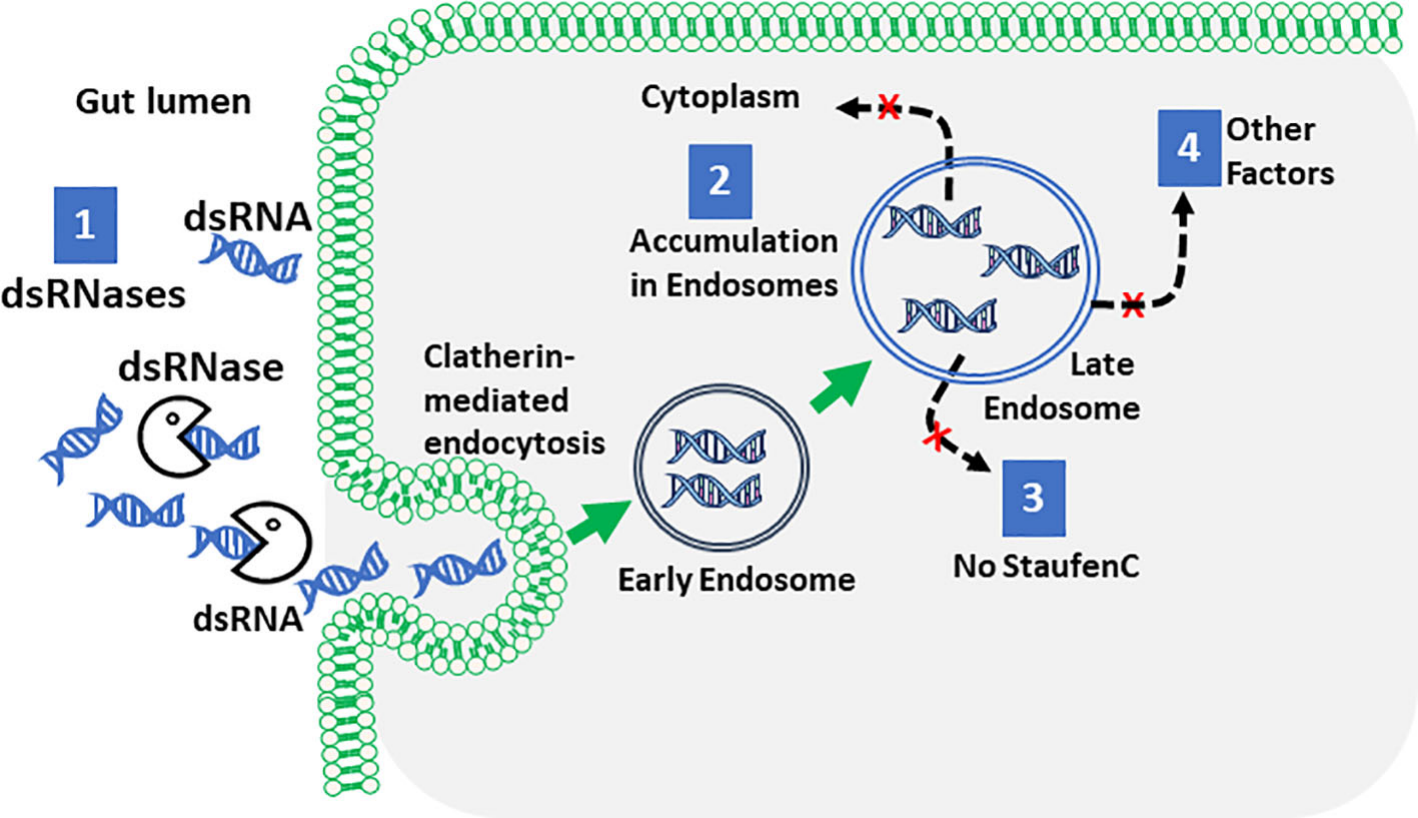
The major roadblocks to efficient RNAi in insects. The cartoon illustrates dsRNA degradation by dsRNases, endosomal entrapment and lack of key players in RNAi such as StaufenC as the major reasons for inefficient RNAi Lepidopterans and other insects.

The first roadblocks encountered by the dsRNA en route to its site of action are double-stranded ribonucleases (dsRNases) (155–160) (**Figure 1**). In *Locusta migratoria*, injection but not oral delivery of dsRNA induces RNAi (151). dsRNA digestion by nucleases is a major contributor to inefficient RNAi (161). Silencing of dsRNase genes improves RNAi efficiency (156, 162–164). dsRNases are more active in the tobacco budworm (*Heliothis virescens*, inefficient RNAi) than those in *L. decemlineata* (165). dsRNA added to the medium of Sf9 cells is degraded by dsRNases secreted by these cells; silencing dsRNases improve RNAi efficiency in these cells (166). RNAi efficiency-related nuclease (REase) identified in lepidopterans has been proposed as a main factor contributing to inefficient RNAi in these insects (157). Formulations that protect dsRNA from nucleases might improve RNAi efficiency in insects that are refractive to dsRNA.

The second roadblock dsRNA encounters while reaching its site of action in the cytoplasm of the cells is crossing the cell membrane and intracellular transport (**Figure 1**). Clatherin-mediated endocytosis was identified as a major pathway for dsRNA uptake (167–174). Macropinocytosis was identified as the major route of dsRNA uptake in the boll weevil (*Anthonomus grandis*) (175). Depending on the concentration and size, dsRNA may enter cells through multiple mechanisms (171). In *L*. *migratoria*, dsRNA does not induce RNAi in follicle cells and oocytes because these tissues do not take up dsRNA (176). In coleopterans, intracellular transport is efficient in delivering dsRNA to the cytoplasm (165). In contrast, in lepidopterans, the intracellular transport is inefficient and most of the dsRNA is trapped in the endosomes (165, 177).

Differences in the expression levels of key players involved in the intracellular transport of dsRNA and its processing to siRNA and recruitment to RISC complex influence RNAi efficiency. Differences in the processing of dsRNA to siRNA were detected between *L. decemlineata* and *S. frugiperda* (165). In insects belonging to orders Lepidoptera, Orthoptera, Hemiptera and Diptera, the processing of dsRNA to siRNA is not as efficient as in coleopterans (178). Differences in the expression levels of Dicer enzymes and their activity could contribute to the differences observed in dsRNA to siRNA processing. The number of Argonaut genes identified, as well as participation of these in siRNA, miRNA and piRNA pathways, vary among insects (172, 179–185). Transgenic expression of *Ago2* in *B. mori* improved RNAi efficiency, suggesting that this protein may be one of the limiting factors responsible for the refractiveness of lepidopterans to RNAi (186).

A double-stranded RNA binding protein (StaufenC) was identified as a key player for efficient RNAi in coleopterans, *L. decemlineata* (65) and *T. castaneum* (187). Interestingly, this gene has been identified only in coleopterans and is required for processing dsRNA to siRNA in these insects. *L. decemlineata*, Lepd-SL1 RNAi resistant cells selected by exposure to dsRNA trigger for multiple generations express lower levels of StaufenC than RNAi susceptible cells. These studies showed that StaufenC is a key player in efficient RNAi coleopterans and a potential target for resistance development to RNAi-based pesticides (187). Recent studies showed that StaufenC functions in coleopterans like Loquacious in *D. melanogaster* which is involved in processing dsRNA to siRNA (188). The nematode *C. elegans* requires systemic RNA interference defective protein 1 (CeSid1) for successful RNAi (189). The expression of CeSid1 in insect cell lines improves RNAi (190–192). However, the requirement of SID1 homologs for RNAi function varies among insects tested (193, 194). Expression of CeSid1 in two *S. frugiperda* cell lines, ovarian-derived Sf9 and midgut-derived Sf17 cells, showed that CeSid1 increases RNAi efficiency in Sf9 but not in Sf17 cells (195). In Sf9 cells expressing CeSid1, decreased accumulation of dsRNA in late endosomes and increased processing dsRNA to siRNA was observed. The Verson’s glands showed the greatest improvement, while the midgut showed the least improvement in RNAi efficiency in CeSID1-expressing transgenic insects. These data point to the variability of RNAi machinery among cell and tissue types (195).

## 7 Strategies to improve RNAi efficiency in major pests and disease vectors

**Figure 2:**
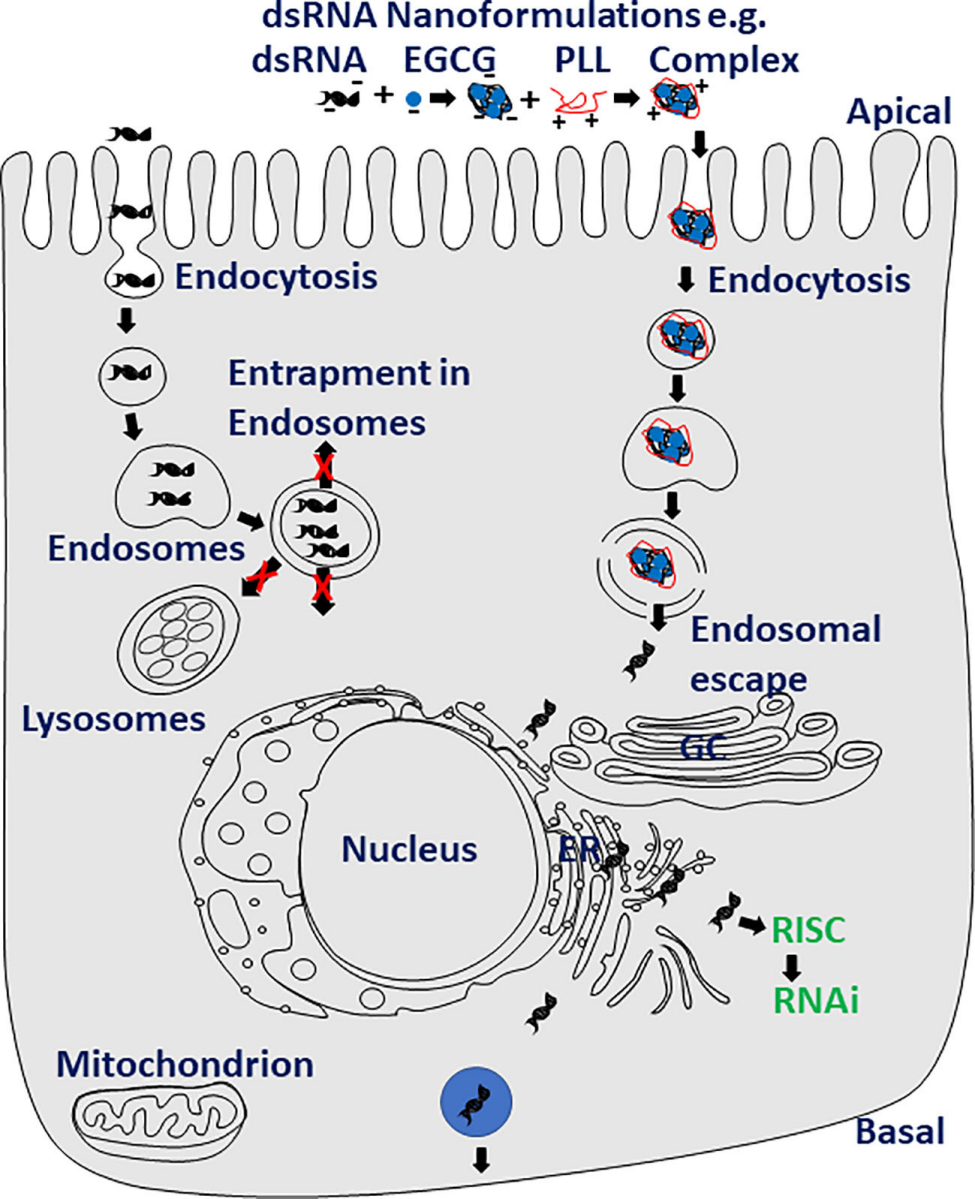
Nanotechnology may help increasing RNAi efficiency in major pests. Nanoformulations of dsRNA such as dsRNA, polyphenol (−)-epigallocatechin-3-O-gallate (EGCG) and poly-l-lysine (PLL) protect dsRNA from degradation by dsRNases, improve uptake, intracellular transport and endosomal escape of dsRNA resulting in increased RNAi efficiency.

Delivery of dsRNA to the site of action in the cytoplasm of the cell will likely improve RNAi efficiency in major pests and disease vectors that are refractive to RNAi. As with any other insecticide-active ingredients, the formulation is key to the successful delivery of dsRNA to the site of action. Several materials were developed to formulate dsRNA with the goal of improving RNAi efficiency (**Figure 2**). In the mosquito, *Anopheles gambiae*, larvae, chitosan and dsRNA formulations improved RNAi efficiency (196). Several other nanoformulations of dsRNA, including guanidine-containing polymers, nanocarrier/dsRNA/detergent formulation, guanylated polymers and branched amphiphilic peptide bilayer conjugated gold nanoparticles were also shown to improve RNAi (175, 197–215). The nanoformulations of dsRNA help protect dsRNA from dsRNases (210–214), facilitate dsRNA penetration through the body wall (216), and improve the cellular uptake and endosomal escape of dsRNA (210–214, 217, 218), ultimately resulting in increased RNAi efficiency (**Figure 2**). Short (23 nt) RNAs with partially closed ends (pcRNAs) were shown to enter cells through a clathrin-independent pathway and improve knockdown of target genes (219). The pcRNAs might help in overcoming some of the hurdles faced by dsRNA reaching the cytoplasm in insects that are recalcitrant to RNAi. dsRNA expressed in microorganisms such as bacteria (117, 220–223), yeast (118, 224–226), algae (119–121), *Bacillus thuringiensis* bacteria (227, 228) and plant viruses (229) were shown to induce knockdown of target genes in insects that ingest these microorganisms expressing dsRNA. Plant-mediated delivery of dsRNA is another option. Indeed, the first RNAi commercial product is based on this method; transgenic maize expressing dsRNA targeting the *SNF7* gene of corm rootworm protects plants from its damage (101). In-planta expression of dsRNA has been demonstrated to protect crops from pests (104, 109, 111, 230–232).

## 8 Conclusions and future perspectives

Over the past 25 years, RNAi made irrefutable contributions to advances in our understanding of insect structure and function. However, advances in developing RNAi methods for pest and disease vector control are not that encouraging; only one commercial product and a few additional products are in the pipeline. One of the major roadblocks to the widespread use of RNAi technology in insect control is its variable efficiency among major insect pests and disease vectors. Also, the availability of transgenic crop plants expressing *B. thuringiensis* toxins that are effective against Lepidopteran pests and inefficient RNAi in major sucking pests slowed the development of RNAi-based products. The potential development of pest resistance to RNAi products is also a major concern. Nanoformulations of dsRNA could help deliver dsRNA to the site of action and improve RNAi efficiency. Advances in methods for producing and delivering dsRNA through microorganisms are encouraging. The research on RNAi during the next 25 years will likely find solutions to these problems and promote widespread use of this technology in insect control and other fields.

## Author contributions

SP confirms being the sole contributor of this work and has approved it for publication.
